# Biofilm Inhibitory Abscisic Acid Derivatives from the Plant-Associated Dothideomycete Fungus, *Roussoella* sp.

**DOI:** 10.3390/molecules23092190

**Published:** 2018-08-30

**Authors:** Chayanard Phukhamsakda, Allan Patrick G. Macabeo, Kamila Tomoko Yuyama, Kevin David Hyde, Marc Stadler

**Affiliations:** 1 Center of Excellence in Fungal Research, Mae Fah Luang University, Chiang Rai 57100, Thailand; chayanard91@gmail.com (C.P.); kdhyde3@gmail.com (K.D.H.); 2 Department of Microbial Drugs, Helmholtz Centre for Infection Research and German Centre for Infection Research (DZIF), partner site Hannover/Braunschweig, Inhoffenstrasse 7, 38124 Braunschweig, Germany; agmacabeo@ust.edu.ph (A.P.G.M.); kamilatomoko@gmail.com (K.T.Y.); 3 Laboratory for Organic Reactivity, Discovery and Synthesis (LORDS), Research Center for the Natural and Applied Sciences, University of Santo Tomas, 1015 Manila, Philippines

**Keywords:** Anti-biofilm, Ascomycota, Pleosporales, structure elucidation

## Abstract

*Roussoella* species are well recorded from both monocotyledons and dicotyledons. As part of a research program to discover biologically active compounds from plant-associated Dothideomycetes in Thailand, the strain *Roussoella* sp. (MFLUCC 17-2059), which represents an undescribed species, was isolated from *Clematis subumbellata* Kurz, fermented in yeast-malt medium and explored for its secondary metabolite production. Bioassay-guided fractionation of the crude extract yielded the new abscisic acid derivative, roussoellenic acid (**1**), along with pestabacillin B (**2**), a related congener, and the cyclodipeptide, *cyclo*(*S*-Pro-*S*-Ile) (**3**). The structure of **1** was determined by 2D NMR spectroscopy and HR-ESIMS data analysis. Compounds **1** and **2** showed inhibitory activity on biofilm formation by *Staphylococcus aureus*. The biofilm formation of *S. aureus* was reduced to 34% at 16 µg/mL by roussoellenic acid (**1**), while pestabacillin B (**2**) only showed 36% inhibition at 256 µg/mL. In addition, compound **1** also had weak cytotoxic effects on L929 murine fibroblasts and human KB3-1 cancer cells.

## 1. Introduction

With over sixty thousand estimated species, the phylum Ascomycota is known to be the most diverse group in the fungal kingdom [1,2,3]. While many studies have reported the richness of biologically active secondary metabolites in this group of fungi, little is known on natural products produced by plant-associated Ascomycota, especially the endophytes and saprobes present in vascular plants [4,5,6].

**Figure 1:**
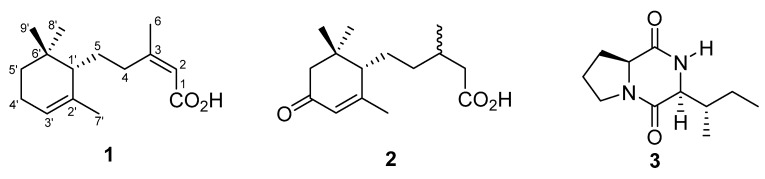
Abscisic acid derivatives and cyclodipeptide from *Roussoella* sp. MFLUCC 17-2059.

Roussoellaceae Liu et al. can be distinguished from other families of Pleosporales (class Dothideomycetes, Ascomycota) by its morphology and is well-resolved in phylogenetic analyses [7]. At present, there is a limited knowledge on the secondary metabolite production of these genera and thus studies are warranted to probe chemical constituents with promising biological activity. The first chemical study on *Roussoella hysterioides* has recently led to the discovery of two novel tetracyclic fusicoccanes, which are oxidized diterpenoid congeners with unique bent structural frameworks [8]. One of these compounds, roussoellol B showed inhibition against the phytopathogenic fungus, *Cochliobolus miyabeanus*. Follow up investigations on two additional species, *R. japanensis* KT1651 and *Roussoella* sp. DLM33, yielded two highly oxidized xanthone derivatives [9] and a dichlorinated polyketide, respectively [10]. During our exploration of new Dothideomycetes in Thailand, a *Roussoella* sp. (MFLUCC 17-2059) was isolated and identified from the decaying parts of *Clematis subumbellata* Kurz (Ranunculaceae). As part of a research program to identify biologically active constituents from Dothideomycetes, we herewith report the isolation, structure elucidation and biological activity of a new abscisic acid derivative (**1**) along with two known fungal metabolites (**2**–**3**) (Figure 1).

## 2. Results and Conclusions

*Roussoella* sp. was isolated from a dried branch of *Clematis subumbellata* Kurz collected in Chiang Rai, Thailand in 2017. The fungus formed cream to yellowish colonies on MEA. The morphological characters of strain MFLUCC 17-2059 resemble those of *Roussoella mangrovei.* However, the results of a multigene phylogenetic analysis suggested that the fungus represents a distinct species. A formal taxonomic description of *Roussoella* sp. will be published elsewhere.

Submerged fermentation of *Roussoella* sp. MFLUCC 17-2059 in shake flask batches on yeast-malt medium (pH 6.3) resulted in a luxuriant growth, and entirely consuming glucose after seven days at 25 °C. The pooled broth extracts were extracted with ethyl acetate to recover the organic metabolites. The crude extracts from submerged cultures showed antimicrobial activities against *Bacillus subtilis*, therefore the extracts were subjected to a bioactivity-guided fractionation using reversed-phase HPLC. *B. subtilis* was selected as the indicator organism, leading to the isolation of the new metabolite roussoellenic acid (**1**). The crude extract was fractionated on reversed-phase HPLC yielding eighteen fractions. Three of those (No. 6, 17, and 18) contained small molecules (LC-MS) and were further purified by RP-HPLC to yield three compounds in quantities sufficient for full spectroscopic characterization.

Roussoellenic acid (**1**) was isolated as a colorless syrup after repeated reversed-phase HPLC purification of fraction seventeen. Its UV spectrum was characteristic of an α,β-unsaturated carboxylic acid with absorption maxima at 218 nm (log ε = 4.1). The molecular formula of **1** was deduced from its protonated molecular ion peak at *m*/*z* 237.1853 [M + H]^+^ by high-resolution mass spectrometry (HRMS; Δmmu + 0.4) as C_15_H_24_O_2_ and further substantiated by ^13^C-NMR spectroscopic data. Further ESI-MS/MS fragmentation of this ion signal resulted in two abundant diagnostic ions: *m*/*z* 191 suggested loss of formic acid (Δ*m*/*z* 46), and *m*/*z* 151 attributed to the *α*-fragmentation with cleavage of the olefinic side chain of the abscisic acid backbone. The formula indicated four indices of hydrogen deficiency.

The ^1^H-NMR spectrum of **1** in MeOH-*d*_4_ featured singlet signals corroborated to four methyl groups [δ_H_ 0.89 (3H, s; CH_3_-9′), 1.00 (3H, s; CH_3_-8′), 1.72 (3H, d, 1.5 Hz; CH_3_-7′), 1.90 (3H, s; CH_3_-6], eight methylene protons [δ_H_ 1.14 (1H, dd *J* = 7.1, 6.0 Hz; H-5′b), 2 × 1.48 (2 × 1H, m; H-5b and H-5′a), 1.61 (1H, m; H-5a), 1.97 (2H, m; H-4′), 2.48 (1H, ddd, 4.9, 10.5, 11.5 Hz; H-4b) and 2.82 (1H, ddd, 4.9, 10.5, 11.5 Hz Hz; H-4a)], an aliphatic methine proton [δ_H_ 1.50, m; H-1′] and two signals of alkenyl protons [δ_H_ 5.31 (1H, brs; H-3′) and 5.62 (1H, s; H-2). The ^13^C-NMR spectrum revealed fifteen carbon resonances, consisting of four methyl, four methylene, one sp^3^ methine, two olefinic methine, one sp^3^ quaternary, two olefinic quaternary, and one carboxyl group. 

**Figure 2:**
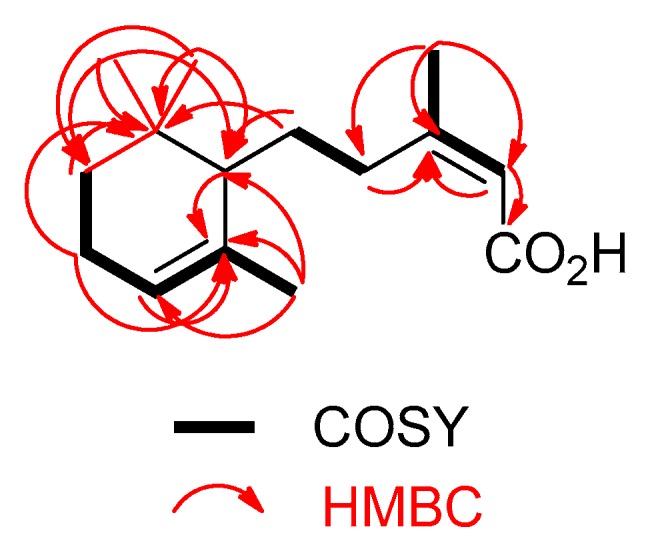
COSY and key HMBC correlations in **1**.

The constitution of structure **1** was unambiguously established through homonuclear and heteronuclear correlation experiments such as proton–proton Correlation Spectroscopy (COSY) and Heteronuclear Multiple Bond Correlation (HMBC). All long-range correlations are summarized in Table 1, and structurally relevant correlations of the COSY and HMBC spectra are presented in Figure 2. These structural characteristics resembled a dihydro-*α*-ionylidene skeleton. In the COSY spectrum, three distinct spin sets of C-2−C-6 (allylic), C-4−C-5−C-1′ and C-7′(allylic)−C-2′−C-3′−C-4′−C-5′ were observed. A complete assignment of all protons and carbons and the establishment of their connectivities were obtained using the HMBC (Figure 2) and Heteronuclear Single Quantum Coherence (HSQC)data. Downfield shifts of H-4b (δ_H_ 2.48) and H-4a (δ_H_ 2.82, m), together with the HMBC correlations between a carboxyl carbon at δ_C_ 169.8 and H-2, H-4a, and H-4b, corroborated the location of the carboxylic acid moiety at C-1. HMBC connections of H-1′ (δ_H_ 1.50) with C-2′ (δ_C_ 133.7), C-6′ (δ_C_ 33.7), C-8′ (δ_C_ 28.2) and C-9′ (δ_C_ 28.1), of H-8′ (δ_H_ 1.00) and H-9′ (δ_H_ 0.89) with C-5′ (δ_C_ 32.8), C-6′, and C-1′ (δ_C_ 50.9), respectively, established the gross sesquiterpenoid structure of **1**. The carbon skeleton is related to abscisic acid [11] and represents a highly reduced derivative through the absence of oxidized functionalities such as a tertiary alcohol at C-1′ and a carbonyl of a ketone at C-4′, and the loss of a double bond at C-4 and C-5.

The conformation of compound **1** was assigned by analysis of its 2D Nuclear Overhauser Effect SpectroscopY (NOESY) data. A cross-peak between resonances assigned to H-5a/H-5b and H_3_-8′, and *pseudo*-axial oriented H-1′ and H_3_-9′ were evident in the spectrum. The 2*Z* configuration was assigned based on the cross-peak between H-2 and H_3_-6. With a substructure akin to the 1,5,5,6-tetrasubstituted cyclohexene of *α*-ionone derivatives and where C-1′ is chiral, the absolute configuration of this stereocenter in **1** can be easily assigned or correlated. In a study conducted by Soorukram and Knochel [12], the asymmetric synthesis of optically active *α*-ionone (incorporating an alkenone substituent at C-1′) and its aliphatic synthetic precursors was achieved via copper-catalyzed anti-S_N_2′ reactions of functionalized organozinc substrates. Accordingly, whether the substituent is conjugated (as in (*R*)-*α*-ionone) or aliphatic (as in (*R*)-dihydro-*α*-ionone), the sign of the specific rotation value remains the same and highly influenced by the chirality present in C-1′. Thus with a levorotatory value, the absolute configuration of C-1′ is proposed to be *S*. The structure of **1** was first reported during a synthetic study of abscisic acid derivatives [13]. However, the characterization data are ambiguous and no NMR and MS spectroscopic data were presented.

Pestabacillin B (**2**) [14] and *cyclo*(*S*-Pro-*S*-Ile) (**3**) [15] were identified by comparing their spectroscopic data with values reported in the literature. 

**Scheme 1:**
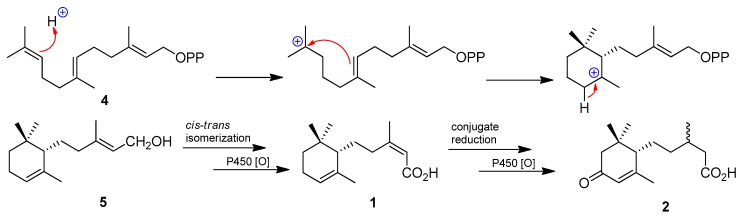
Putative biogenetic pathway of **1** and **2**.

Abscisic acid and its derivatives have been detected in culture filtrates of selected saprophytic and plant pathogenic fungi [16]. However, reduced congeners such as **1** and **2** are rare. Interestingly, compound **2** was produced as a result of a co-culture experiment between the endophytic fungus *Pestalotiopsis microspora* and *Bacillus subtilis* [14]. Based on a previously reported biochemical data outlining the biosynthetic pathways *en route* abscisic acids [17], the cascades entail direct cyclization of farnesyl diphosphate (**4**) and relay of oxidation reactions. Thus biosynthetically, **1** could be derived after the carbocyclization of **4** via stereoselective ring closure of C-6′ and C-1′ to afford dihydro-*α*-ionylidenethanol **5**, *cis*-*trans* isomerization in the conjugated olefin (C-2–C-3), and oxidation of primary alcohol (C-1) to carboxylic acid. Reduction of C-2 olefin in **1** followed by P450 monooxygenase [18] driven C-4 allylic oxidation produces compound **2** (Scheme 1).

The purified compounds were tested for their antimicrobial activities against various organisms (Table 2). Roussoellenic acid (**1**) showed minimum inhibitory concentrations (MIC) being more significantly inhibitory than pestabacillin B (**2**). Compound **1** exhibited weak antibacterial activity against *Bacillus subtilis*, *Micrococcus luteus*, and *Staphylococcus aureus* (MIC = 66.7 µg/mL). It also exhibited antifungal activity with similar MIC values against *Mucor hiemalis*. The cytotoxicity of compounds **1** and **2** to KB3.1 and L929 cells were also determined for an initial assessment of cancer cell toxicity. Compound **1** exhibited weak cytotoxic effects on L929 murine fibroblasts and human KB3-1 cancer cells (Table 2).

As a rule, all metabolites that are being isolated in our laboratory that showed only moderate to weak antimicrobial and cytotoxic activities, are being subjected to biofilm inhibition assays. The rationale for this procedure is that novel or rare metabolites that have no direct toxic effect on the pathogenic target organisms may still have a potential, e.g., as inhibitors of biofilm formation. As outlined in a recent review [19], and as demonstrated by some of our own recent publications [20,21], several classes of natural products, including fungal metabolites can inhibit the formation of biofilms and thus constitute candidates for combinational therapy as enhancers of the potency of commercial or novel antibiotics.

Thus, roussoellenic acid (**1**) and pestabacillin B (**2**) were evaluated against *Staph. aureus* to determine if they are able to inhibit biofilm formation. Compound **1** inhibited 34% of the biofilm formed by *S. aureus* at 16 µg/mL test concentration, while compound **2** inhibited 36% of the bacterial biofilm at 256 µg/mL (Table 3). As compared to the effects reported in our recent publications. compound **1** actually showed stronger activities than the other inhibitors we found so far, including microporenic acids and sclerin derivatives [19,20]. It is also interesting that compound **2** showed weaker effects despite its structural similarity to **1**. The reasons for this phenomenon remains to be validated in the future. Abscisic acid and some of its derivatives are important hormones in plants. They are involved in the regulation of seed development by controlling desiccation tolerance, storage product deposition, and dormancy [13]. However, to the best of our knowledge, none of the derivatives of abscisic acid has so far been associated with inhibition of biofilm formation in a human pathogen [21]. Our results therefore provide first evidence on how to further pursue this phenomenon.

## 3. Materials and Methods

### 3.1. General Information

Optical rotations were determined using a Perkin-Elmer (Überlingen, Germany) 241 spectrophotometer; ultraviolet (UV) spectra were recorded with a Shimadzu (Duisburg, Germany) UV-2450 UV-Vis spectrophotometer. NMR spectra were recorded on a Bruker (Bremen, Germany) Ascend 700 spectrometer equipped with 5 mm TXI cryoprobe (^1^H-700 MHz, ^13^C-175 MHz) spectrometer. HR-ESI-MS mass spectra were recorded using a Bruker (Bremen, Germany) Agilent 1260 series HPLC-UV/Vis system (column 2.1 × 50 mm, 1.7 m, C18 Acquity UPLC BEH (waters), solvent A: H_2_O + 0.1% formic acid; solvent B: AcCN + 0.1% formic acid, gradient: 5% B for 0.5 min increasing to 100% B in 19.5 min and then maintaining 100% B for 5 min, flow rate 0.6 mL/min, UV/Vis detection at 200–600 nm combined with ESI-TOF-MS (MaXiS, Bruker, Bremen, Germany) [scan range 100–2500 *m*/*z*, capillary voltage 4500 V, dry temperature 200 °C]. Chemicals and solvents were obtained from AppliChem GmbH (Darmstadt, Germany), Avantor Performance Materials (Arnhem, Netherlands), Carl Roth GmbH & Co. KG (Karlsruhe, Germany) and Merck KGaA (Darmstadt, Germany) in analytical and HPLC grade.

### 3.2. Fungal Material

The specimen *Clematis subumbellata* Kurz was collected from Chiang Rai, Thailand by one of the authors (C.P.) in May, 2017. The dried herbarium specimen and culture are deposited at Mae Fah Luang culture collections as MFLU 17-1465 and MFLUCC 17-2059, respectively. The fungus was identified as *Roussoella* sp. by morphological studies and the DNA sequences data (5.8S gene region, the internal transcribed spacer ITS1 and ITS2 and TEF1-alpha). These sequence data are deposited in GenBank with accession number MH744730 and MH75023, respectively. Genomic DNA Miniprep kit (Bio Basic Canada Inc., Markham, ON, Canada). A Precellys 24 homogenizer (Bertin Technologies, Saint-Quentin-en-Yvelines, France) was used for cell disruption at a speed of 6000 rpm for 40 s for two times. The ITS gene region were amplified with primers ITS5 and ITS4, while TEF1-alpha were amplified by using 983F and 2218R. According to the interpretations as a new species (see S1), results will be described in a later taxonomic paper.

### 3.3. Fermentation and Extraction

A well-grown mycelial culture of *Roussoella* sp. (15 days old) on yeast-malt glucose (YMG) agar plates (1% malt extract, 0.4% d-Glucose, 0.4% Yeast extract, 2% agar, pH 6.3) was cut into small pieces using a 7 mm cork borer. Five plugs were transferred into each of the 30 sterilized Erlenmeyer flasks containing liquid YMG media. The cultures were incubated in a dark room at 25 °C on a rotary shaker (140 rpm) for seven days. After the sugar was consumed, fermentation was terminated 3 days after glucose depletion. The mycelia and supernatant were separated using vacuum filtration, and the mycelia were homogenized and extracted with acetone under ultrasonic conditions (3×). The combined acetone extracts were evaporated in vacuo (40 °C), and the crude product was suspended in distilled water and extracted with an equal volume of ethyl acetate (4×). The aqueous layer was discarded and the organic phase dried over anhydrous sodium sulfate. The resulting ethyl acetate extracts were evaporated to dryness to afford 1 g of crude material. The supernatant was mixed with Amberlite XAD-16 N (30 g per 1 L) and extracted according to a previously described procedure [19].

### 3.4. Isolation of ***1***–***3***

The crude extracts from the mycelia and supernatant yielded a yellowish to brown solid residue. The extracts in methanol were filtered on a RP solid-phase cartridge (Strata-X 33 mm, polymeric reversed phase; Phenomenex Aschaffenburg, Germany) to yield 800 mg of crude material. Purification was achieved on a preparative HPLC (Gilson, Middleton, WI, USA) equipped with a GX-271 Liquid Handler, a 172 DAD, and a 305 and 306 pump (with 50SC Piston Pump Head) [22]. A VP Nucleodur 100-10 C18 ec column (150 × 40 mm, 7 μm; Macherey-Nagel) was used as stationary phase. The mobile phase was composed of deionized water (Milli-Q, Millipore, Schwalbach, Germany) with 0.05% trifluoroacetic acid (solvent A) and acetonitrile (AcCN, HPLC-grade) with 0.05% trifluoroacetic acid (solvent B). Separation was carried out according to the following gradients: linear gradient of solvent B from 10% to 100% in 35 min, followed by isocratic conditions at 100% for 10 min, with a flow rate of 35 mL/min. UV detection was carried out at 210, 280 and 354 nm. Fractions were collected and combined according to the observed peaks. Compound **1** (8.2 mg) was obtained at a retention time (t*_R_*) of 36.5–37 min while compounds **2** (12 mg) and **3** (6.2 mg) were yielded at t*_R_* = 37–38 min and t*_R_* = 16.5–17.5 min, respectively. The fractions eluting at t*_R_* = 36.5–37 min were bioactive and contained **1** with some impurity. This fraction was rechromatographed on a Gemini 10u C18 110A column (250 × 21.20 mm, 10 μm) using the following gradients; linear gradient of solvent B from 40% to 100% in 20 min, followed by isocratic conditions at 100% for 20 min, with a flow rate of 15 mL/min. Compound **1** was obtained at t*_R_* = 26−27 min to give a final yield of 2.7 mg.

Roussoellenic acid (**1**): colorless oil; [α${]}_{D}^{25}$ = −106.0 (c 0.1, MeOH); ^1^H-NMR and ^13^C-NMR data, see Table 1; HR-ESIMS [M + H]^+^ *m/z* 237.1853 (calc. 237.1849, C_15_H_25_O_2_); see Figure S8.

### 3.5. Biological Activities

#### Antimicrobial Activity and Cytotoxicity Assays

The MIC of the test compounds against bacteria, yeasts, and filamentous fungi were determined using a serial dilution technique on 96-well microtiter plates [23]. In vitro cytotoxicity (IC_50_) of compounds **1** and **2** were determined against mouse fibroblast L929 and HeLa (KB-3.1) cell lines according to a previously described procedure [24]. The assays for determination of antimicrobial and cytotoxic activities were performed according to our standard protocols, two times with each assay carried out in duplicate regarding the concentration range of samples.

### 3.6. Inhibition of Biofilm Formation

The biofilm forming strain *Staphylococcus aureus* DSM1104 was enriched overnight on a casein-peptone soymeal-peptone (CASO) medium from Sigma-Aldrich (MO, United States of America) containing 4% glucose (pH 7.0). The ability of **1** and **2** to prevent *Staph. aureus* biofilm formation was performed using a previously described protocol [20].

The assay was carried out in 96-well tissue microtiter plates (TPP, Switzerland). The turbidity of the pre-inoculum was adjusted to obtain a 0.5 McFarland standard and the test compounds were serially diluted (1 to 256 µg/mL) and incubated for 20 h at 37 °C. Methanol and CASO medium containing 4% glucose were used as a negative controls and tetracycline (100 µg/mL) was used as the positive control. The biofilm inhibition assay was performed in triplicate. After incubation, biofilm formations were measured after staining with 0.1% crystal violet solution (Fluka, Steinheim, Germany).

## Figures and Tables

**Table 1:** NMR spectroscopic data (MeOH-*d*_4_, 700 MHz) for roussoellenic acid (**1**).

Position	δ_H_^1^	δ_C_^1^	HMBC ^2^
1	-	169.8	-
2	5.62, s	117.2	1, 3, 4, 6
3	-	162.2	-
4a	2.82 (ddd, 11.5, 10.5, 4.9)	35.1	1, 2, 3, 6, 1′
4b	2.48 (ddd, 11.5, 10.5, 4.9)		
5a	1.61 ^3^	30.7	3, 1′, 2′, 6′
5b	1.48 ^3^		
6	1.90, s	25.5	2, 3, 4, 6
1′	1.50 ^3^	50.9	4, 2′, 5′, 6′, 7′, 8′, 9′
2′	-	137.7	-
3′	5.31, brs	121.3	1′, 2′, 4′, 7′
4′	1.97 ^3^	24.1	2′, 3′, 5′
5a′	1.48 ^3^	32.8	1′, 2′, 5′, 8′, 9′
5b′	1.14 (dd, 7.1, 6.0)		
6′	-	33.7	-
7′	1.72, (d, 1.5)	23.8	1′, 2′, 3′
8′	1.00, s	28.2	1′, 5′, 6′, 9′
9′	0.89, s	28.1	1′, 5′, 6′ 8′

^1^ δ values were established from HSQC-DEPT, COSY and HMBC experiments. ^2^ HMBC correlations, optimized for 10 Hz, are from the proton(s) stated to the indicated carbon(s). ^3^ Multiplicity not reported due to overlap of signals.

**Table 2:** Antimicrobial and cytotoxic activities of compounds **1** and **2**.

Strain	Compound 1	Compound 2	Positive Control (µg/mL)	
Bacteria	MIC (µg/mL)			
*Bacillus subtilis* DSM 10	66.7	-	4.1	Oxytetracyclin
*Chromobacterium violaceum* DSM 30191	-	-	0.4	Oxytetracyclin
*Escherichia coli* DSM 1116	-	-	3.3	Oxytetracyclin
*Micrococcus luteus* DSM 1790	66.7	-	0.2	Oxytetracyclin
*Mycobacterium smegmatis* ATCC 700084	-	-	3.3	Kanamycin
*Pseudomonas aeruginosa* PA14	-	-	1.7	Gentamycin
*Staphylococcus aureus* DSM 346	66.7	-	3.3	Oxytetracyclin
Fungi				
*Candida albicans* DSM 1665	-	-	66.7	Nystatin
*Mucor hiemalis* DSM 2656	66.7	-	33.3	Nystatin
*Pichia anomala* DSM 6766	-	-	33.3	Nystatin
*Rhodoturula glutinis* DSM 10134	-	-	33.3	Nystatin
*Schizosaccharomyces pombe* DSM 70572	-	-	33.3	Nystatin
Cell lines	Cytotoxicity IC_50_ (µg/mL)			
KB 3.1 HeLa	14	-	5 × 10^−4^	epothilone B
L929	27	-	1.1 × 10^−3^	epothilone B

(-) no activity; Starting concentration for antimicrobial assay and cytotoxicity assay were 66.7 and 300 µg/mL, respectively.

**Table 3:** Biofilm inhibitory activity of compounds **1** and **2**.

Test Organisms	Compound 1	Compound 2	Tetracycline
	Biofilm Inhibition %	Biofilm Inhibition %	Biofilm Inhibition %
*Staphylococcus aureus* DSM 1104	98% (256 µg/mL)	36% (256 µg/mL)	88% (100 µg/mL)
	83% (128 µg/mL)	23% (128 µg/mL)	
	73% (64 µg/mL)	-	
	61% (32 µg/mL)	-	
	34% (16 µg/mL)	-	

(-) no activity.

## Supplementary Materials

The following are available online: Figures S1.1–1.2, S2–S15, and Table S13.
