# Supplementary material for: Biofilm Inhibitory Abscisic Acid Derivatives from the Plant-Associated Dothideomycete Fungus, Roussoella sp
Source: Molecules. 2018 Aug 30;23(9):2190. doi: 10.3390/molecules23092190 (PMC6225182; doi:10.3390/molecules23092190)
Supplement: Supplementary file 1 [file molecules-23-02190-s001.pdf]

SUPPLEMENTARY MATERIAL FOR

Biofilm Inhibitory Abscisic Acid Derivatives from the Plant-Associated Dothideomycete Fungus, *Roussoella* sp.

Chayanard Phukhamsakda [1], Allan Patrick G. Macabeo [2,3], Kamila Tomoko Yuyama [2], Kevin David Hyde [1] and Marc Stadler [2,*]

[1] Center of Excellence in Fungal Research, Mae Fah Luang University, Chiang Rai, 57100 Thailand; chayanard91@gmail.com; kdhyde3@gmail.com
[2] Department of Microbial Drugs, Helmholtz Centre for Infection Research and German Centre for Infection Research (DZIF), partner site Hannover/Braunschweig, Inhoffenstrasse 7, 38124, Braunschweig, Germany; kamilatomoko@gmail.com
[3] Laboratory for Organic Reactivity, Discovery and Synthesis (LORDS), Research Center for the Natural and Applied Sciences, University of Santo Tomas, 1015 Manila, Philippines; agmacabeo@ust.edu.ph

* Correspondence: Marc.Stadler@helmholtz-hzi.de; Tel.: +49-531-6181-4240.

LIST OF SUPPORTING INFORMATION

	Page
**S1.** Morphological features and molecular phylogenetics	3
**S2.** ^{1}H NMR spectrum (MeOH-d_4, 700 MHz) of roussoellenic acid (**1**)	5
**S3.** ^{13}C NMR spectrum (MeOH-d_4, 175 MHz) of roussoellenic acid (**1**)	5
**S4.** HSQC-DEPT spectrum of I roussoellenic acid (**1**)	6
**S5.** COSY spectrum of roussoellenic acid (**1**)	6
**S6.** HMBC spectrum of roussoellenic acid (**1**)	7
**S7.** NOESY spectrum of roussoellenic acid (**1**)	7
**S8.** LC-DAD-HR-ESIMS spectrum of roussoellenic acid (**1**)	8
**S9.** ^{1}H NMR spectrum ($CDCl_3$, 700 MHz) of pestabacillin B (**2**).	9
**S10.** ^{13}C NMR spectrum ($CDCl_3$, 175 MHz) of pestabacillin B (**2**).	9
**S11.** ^{1}H NMR spectrum (MeOH-d_4, 700 MHz) of *cyclo*(*S*-Pro-*S*-Ile) (**3**).	10
**S12.** ^{13}C NMR spectrum (MeOH-d_4, 175 MHz) of *cyclo*(*S*-Pro-*S*-Ile) (**3**).	10
**S13.** Antimicrobial assay results of compounds **1-3**.	11
**S14.** Cytotoxicity assay results of compounds **1**.	12
**S15.** Biofilm inhibitory activity of compounds **1-3.**	13

S1. Morphological features and molecular phylogenetic

S1.1 Identification of *Roussoella sp.* MFLUCC 17-2059

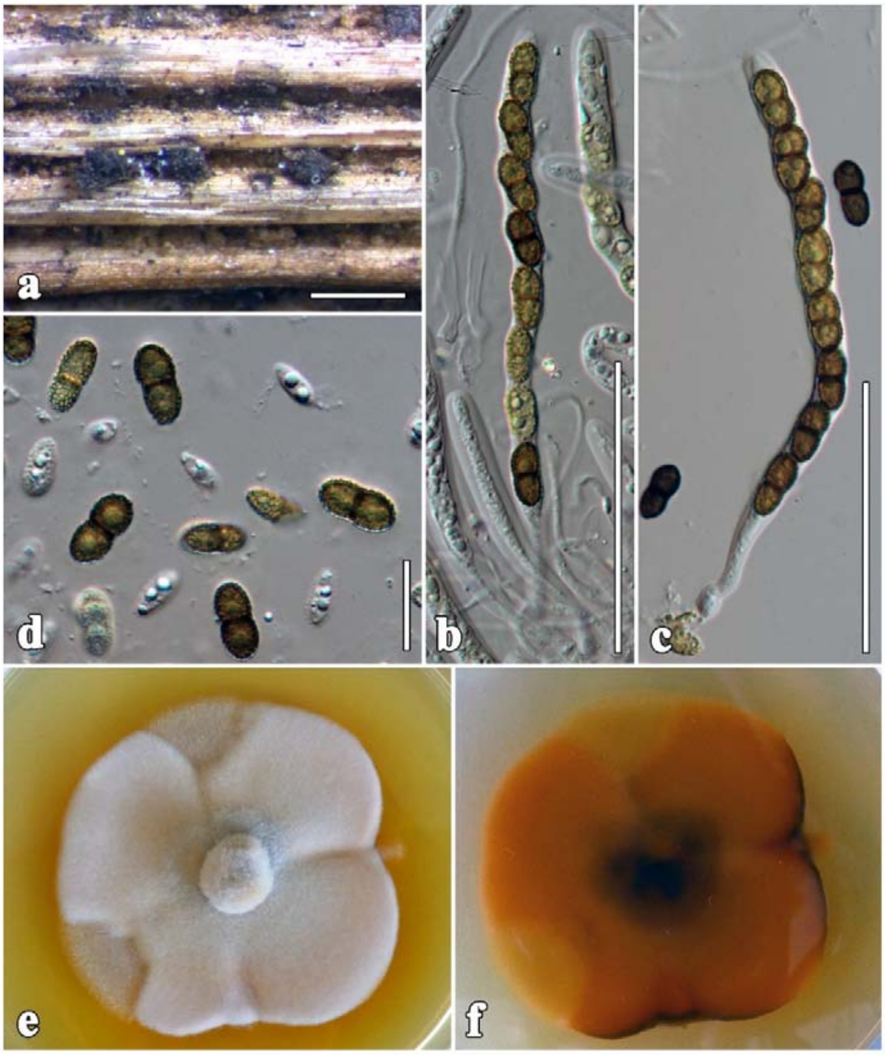

Figure S1.1 *Roussoella sp.* MFLUCC 17-2059 **a** Appearance of ascomata on host surface **b-c** Asci **d** Ascospores e-f Culture characters on YMG agar. **Scale bars:** a = 500 *µm*, b-c = 50 *µm*, d = 10 *µm.*

Material examined: Dried branched *Clematis subumbellata* (Ranunculaceae), Thailand, living culture = MFLUCC 17-2059.

S1.2 Phylogenetic study

The aligned ITS and TEF-1α sequences were concatenated to generate multigenes alignment phylogenetic tree. Maximum Likelihood were constructed by using CIPRES webportal online tools (Miller et al. 2010), including 1,000 bootstrap replicates. According to the data set combining the morphological features and phylogenetic analysis, the fungus was identified as *Roussoellaceae* member.

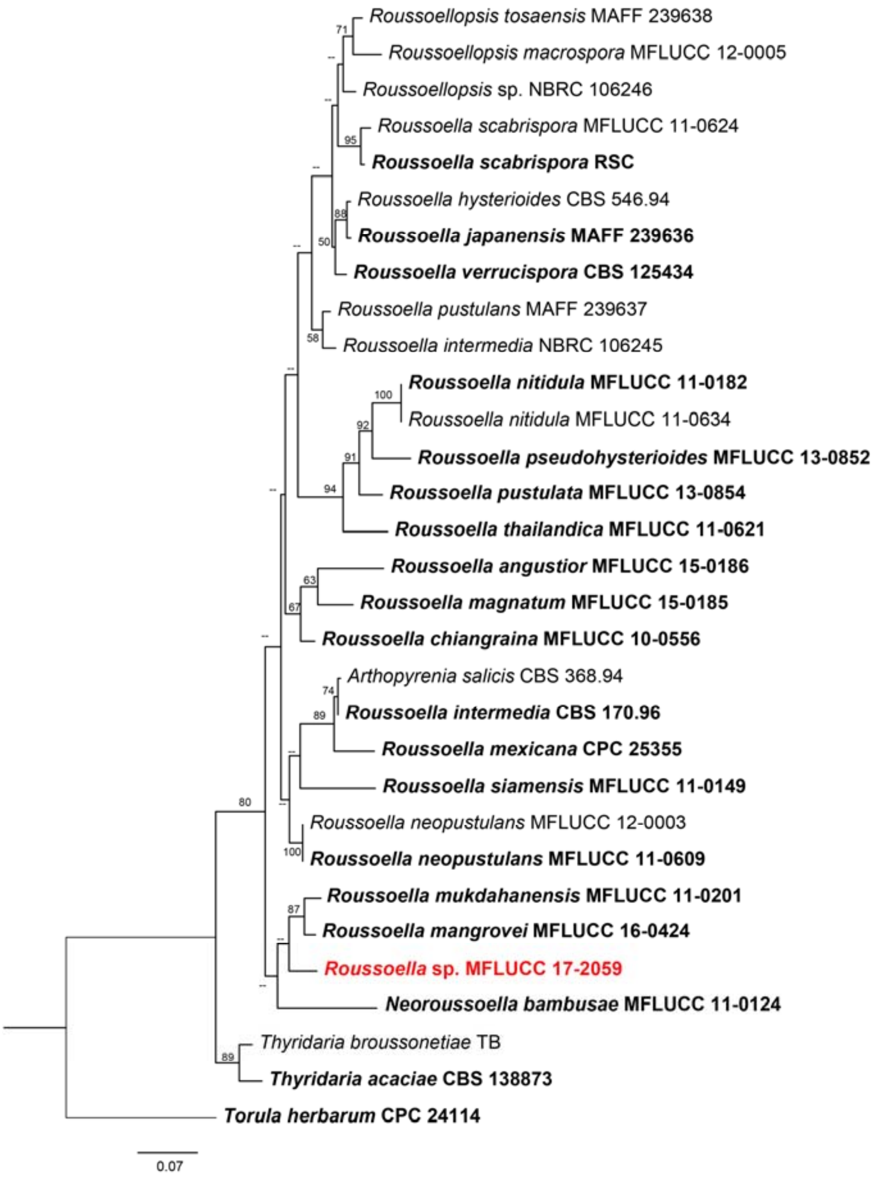

Fig S1.2. Phylogenetic tree of the combined data set of two markers, RAxML (ITS and TEF-1α). The strain produced secondary metabolites in this study is marked in red.

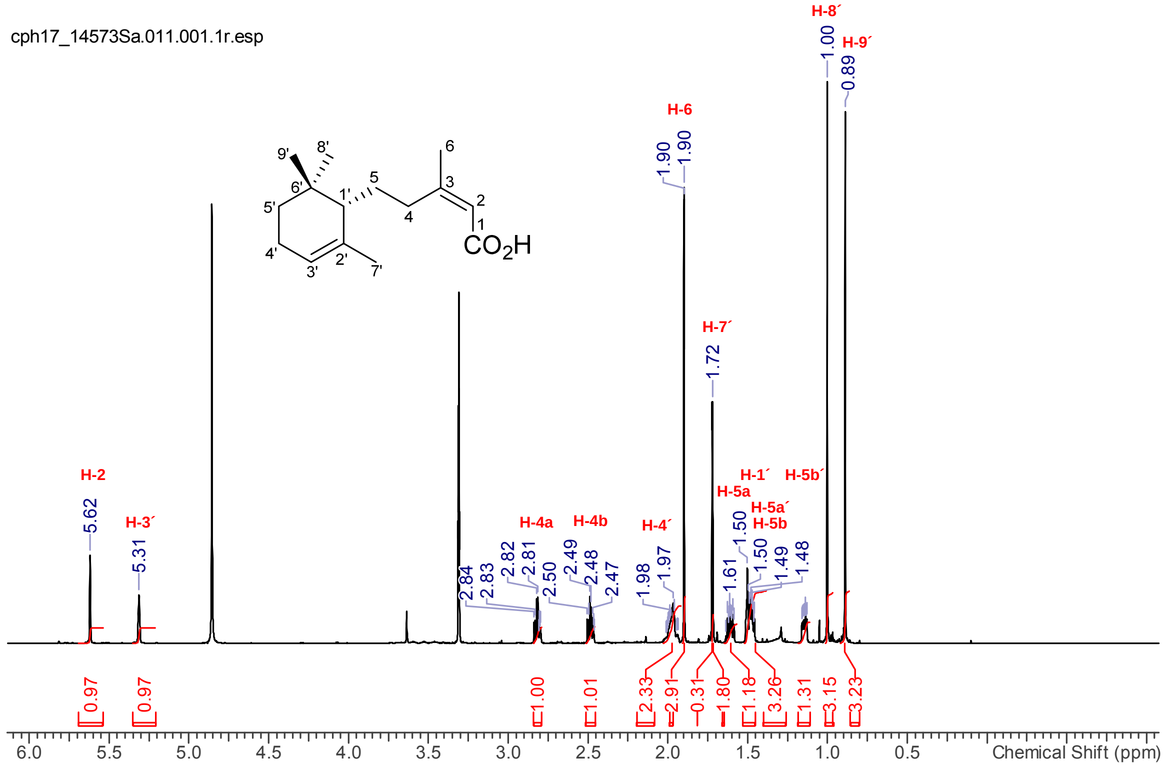

S2. ^{1}H NMR spectrum (MeOH-d_4, 700 MHz) of roussoellenic acid (**1**).

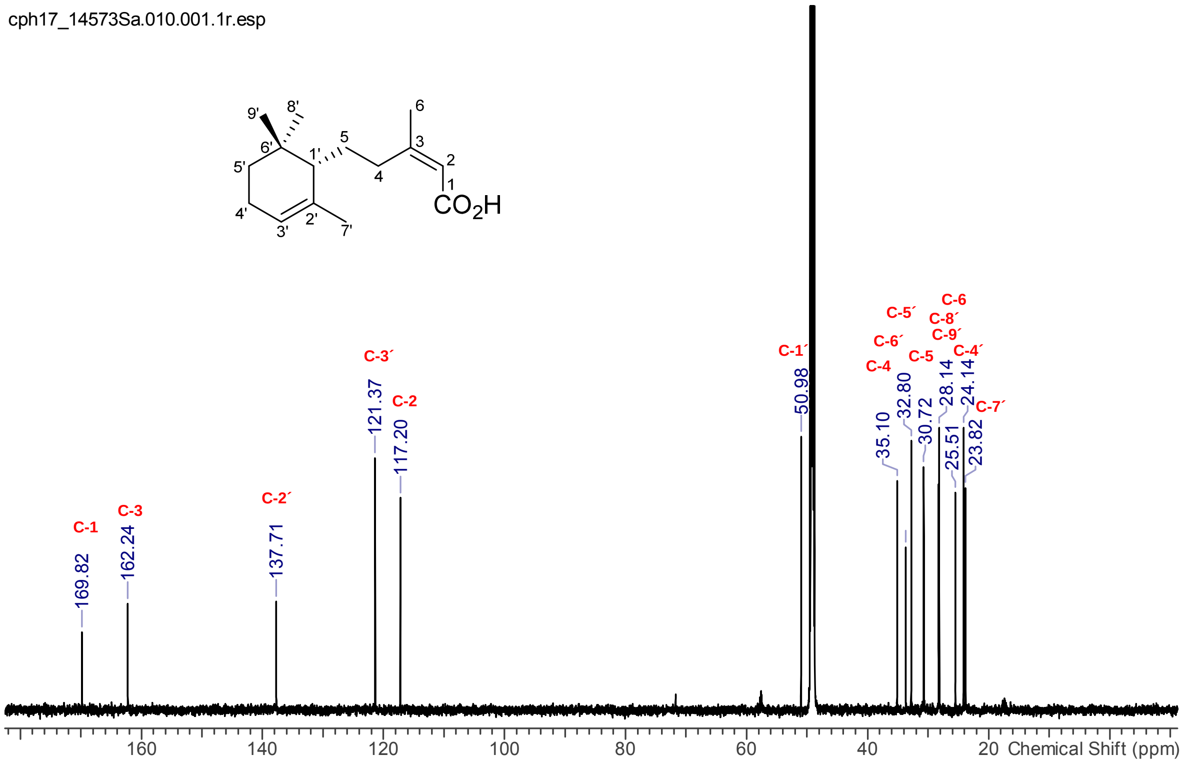

S3. ^{13}C NMR spectrum (MeOH-d_4, 175 MHz) of roussoellenic acid (**1**).

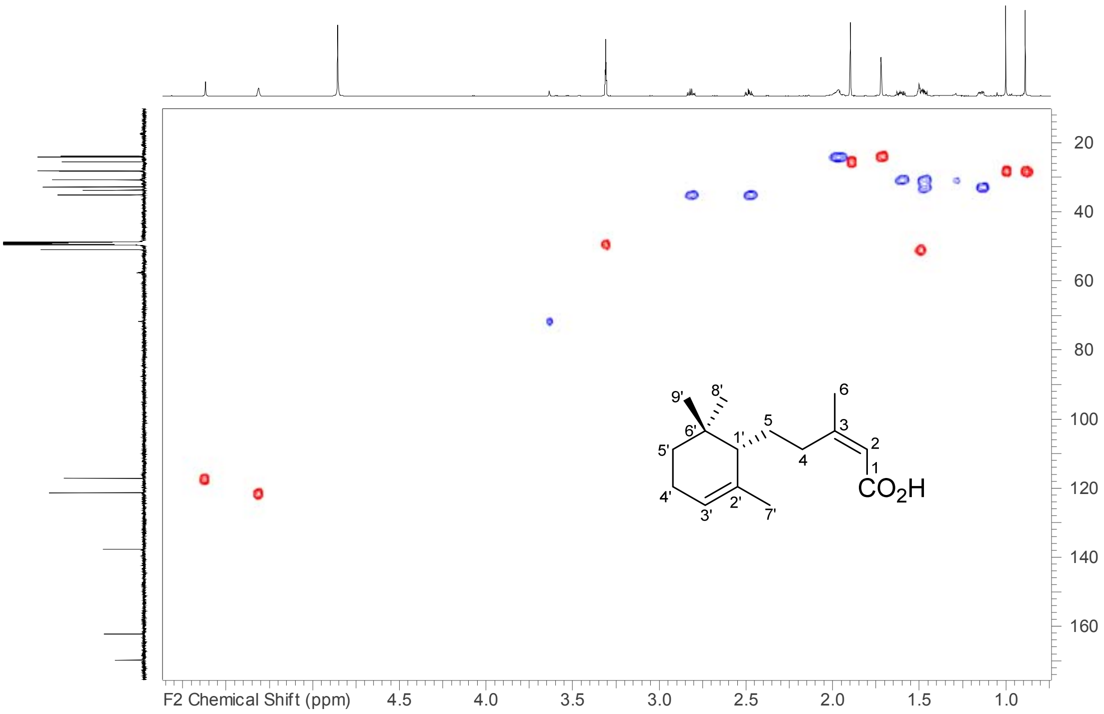

S4. HSQC-DEPT* edited spectrum of roussoellenic acid (**1**).

* blue crosspeak – CH_2; red crosspeak – CH/CH_3

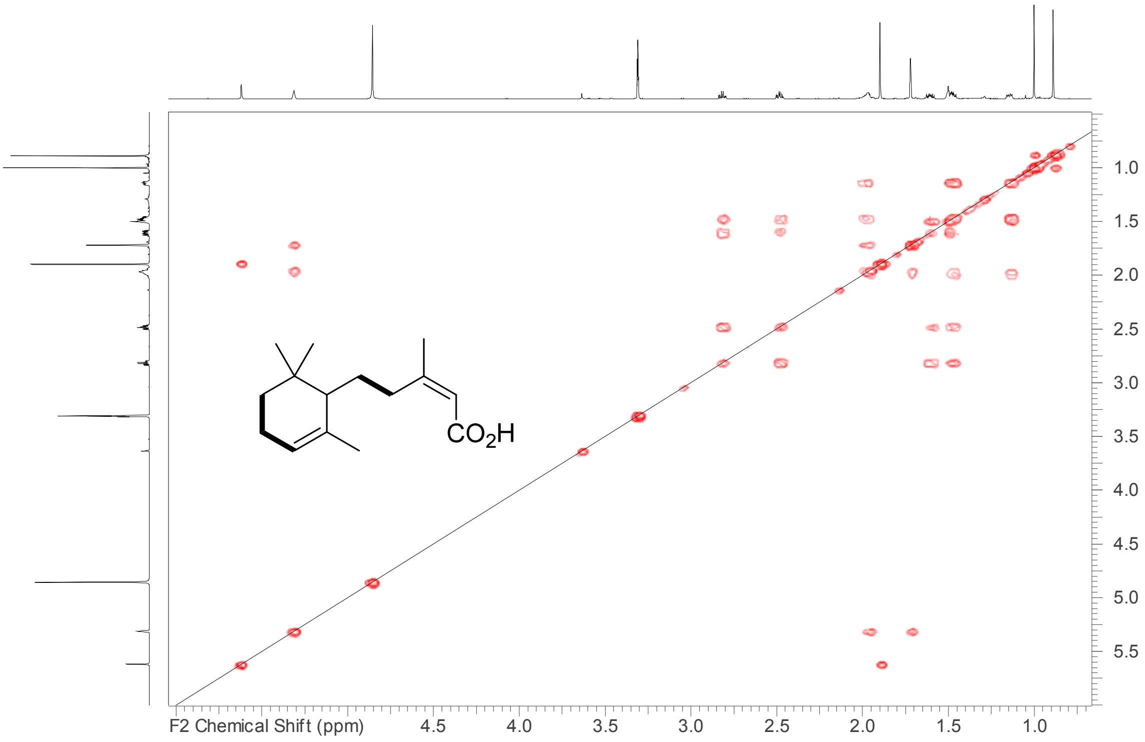

S5. COSY spectrum of l roussoellenic acid (**1**).

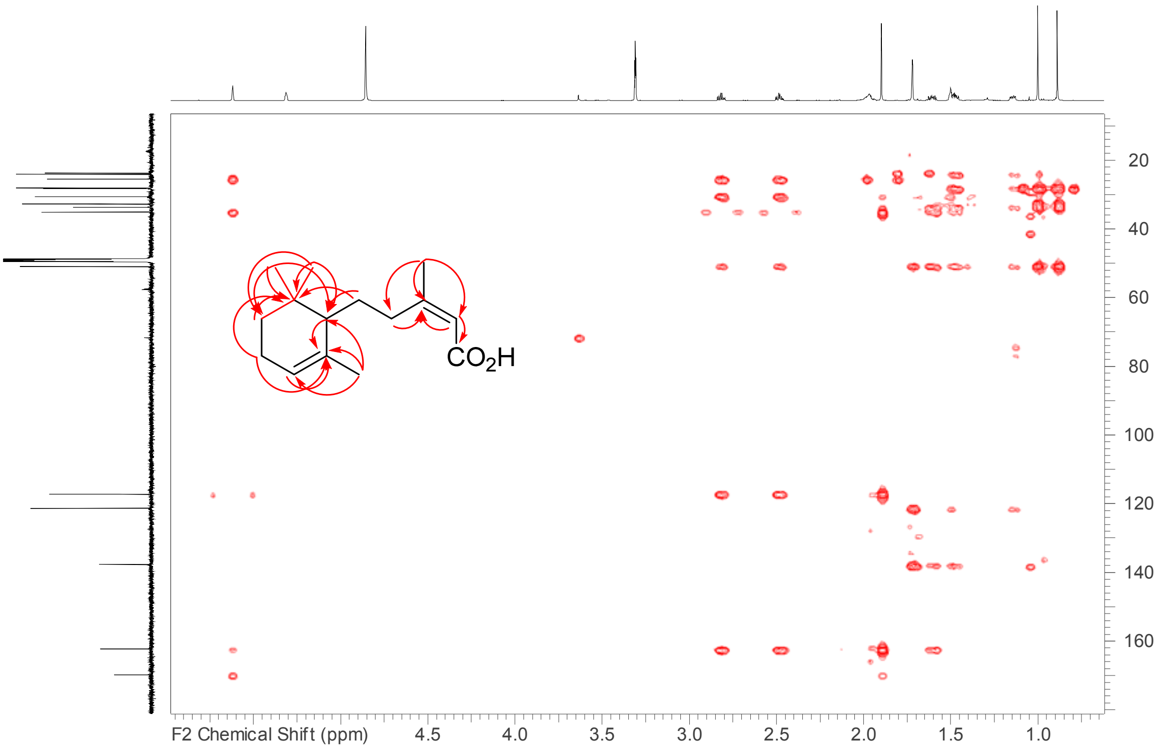

S6. HMBC spectrum of roussoellenic acid (**1**).

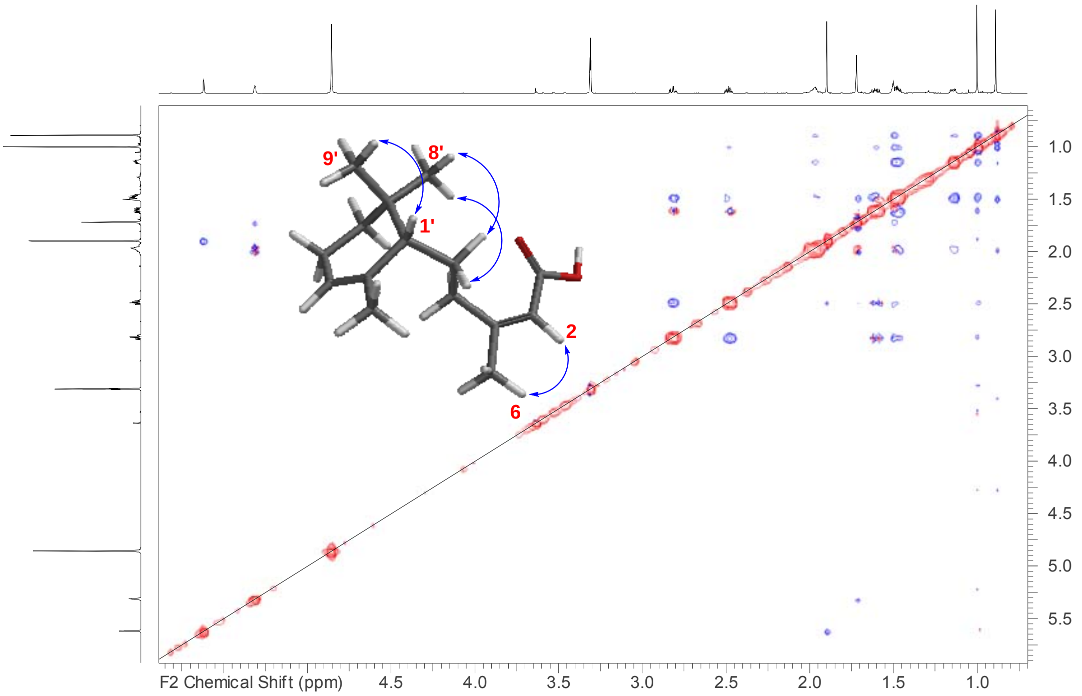

S7. NOESY spectrum of roussoellenic acid (**1**).

* NOE correlations are indicated by blue off-diagonal peaks.

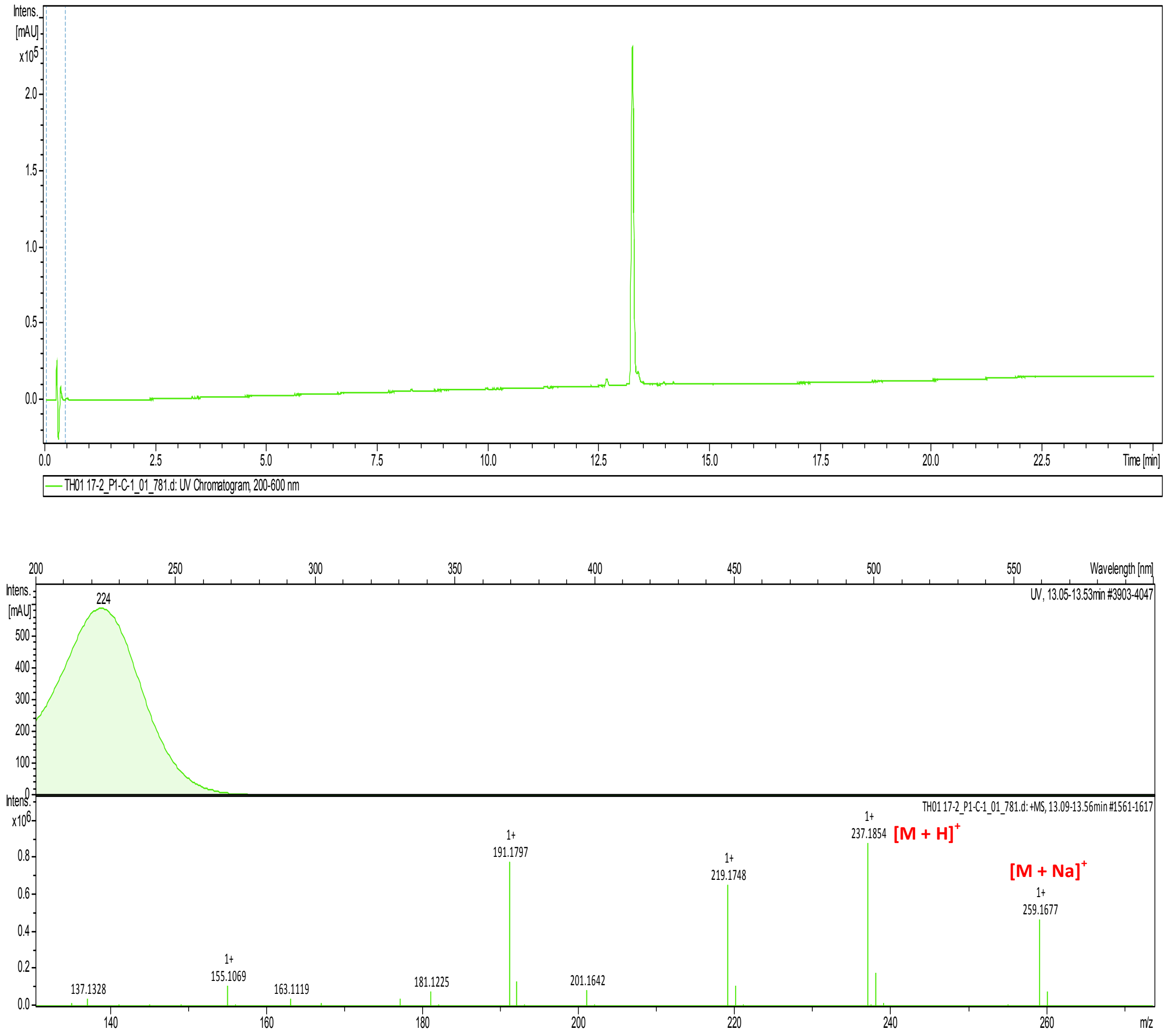

S8. LC-DAD-HR-ESIMS spectrum of roussoellenic acid (**1**).

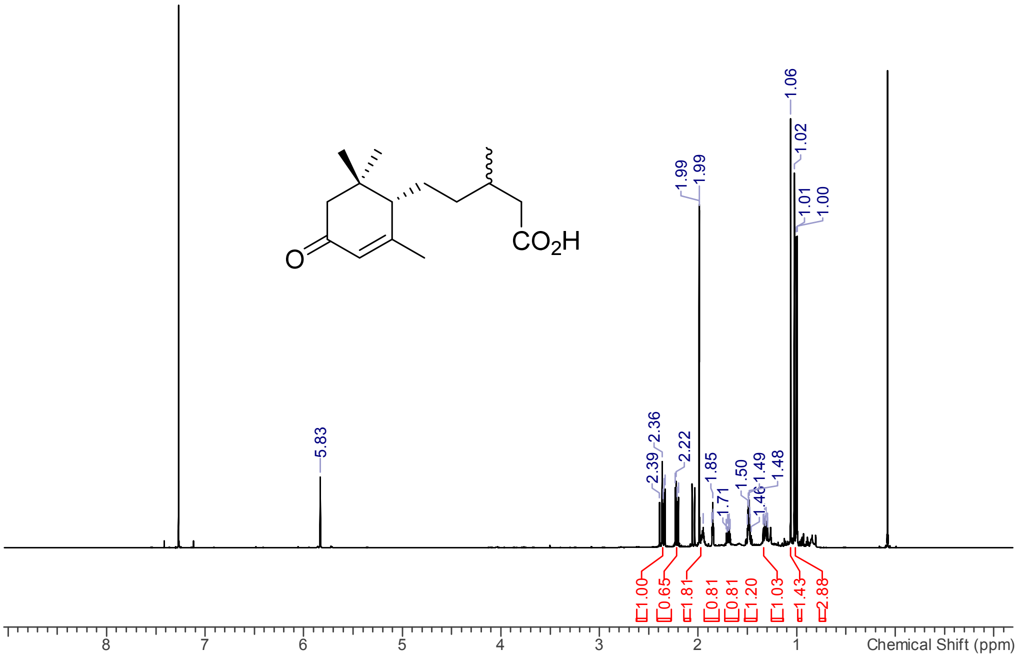

S9. ¹H NMR spectrum ($CDCl_3$, 700 MHz) of pestabacillin B (**2**).

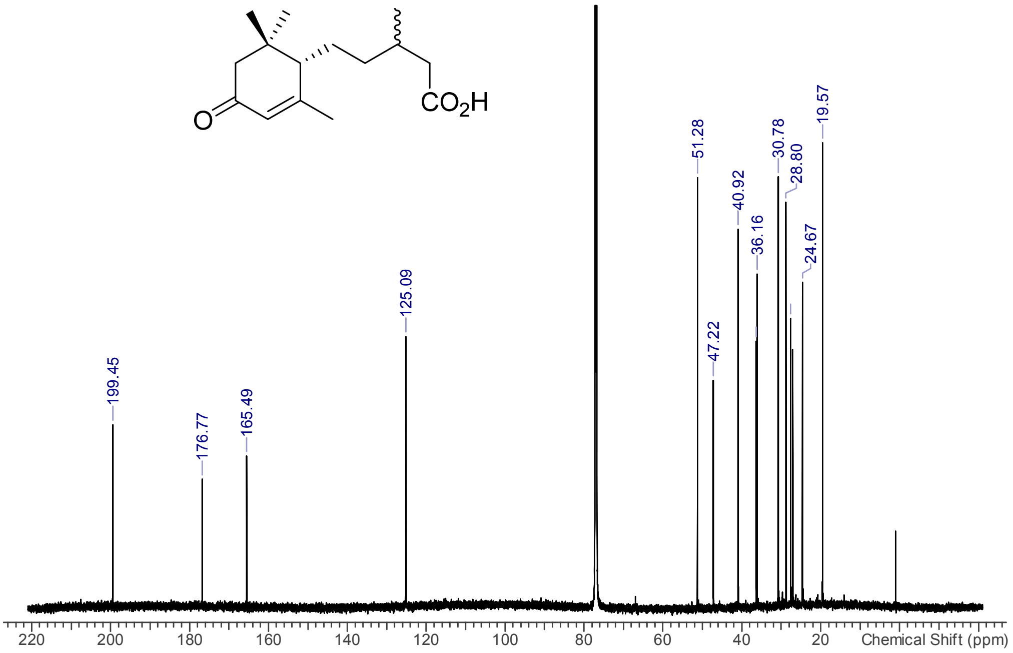

S10. ¹³C NMR spectrum ($CDCl_3$, 175 MHz) of pestabacillin B (**2**).

Liu, S.; Dai, H.; Heering, C.; Jania, C.; Lin, W.; Liu, Z.; Proksch, P. *Tetrahedron Lett.* **2017**, *58,* 257-261.

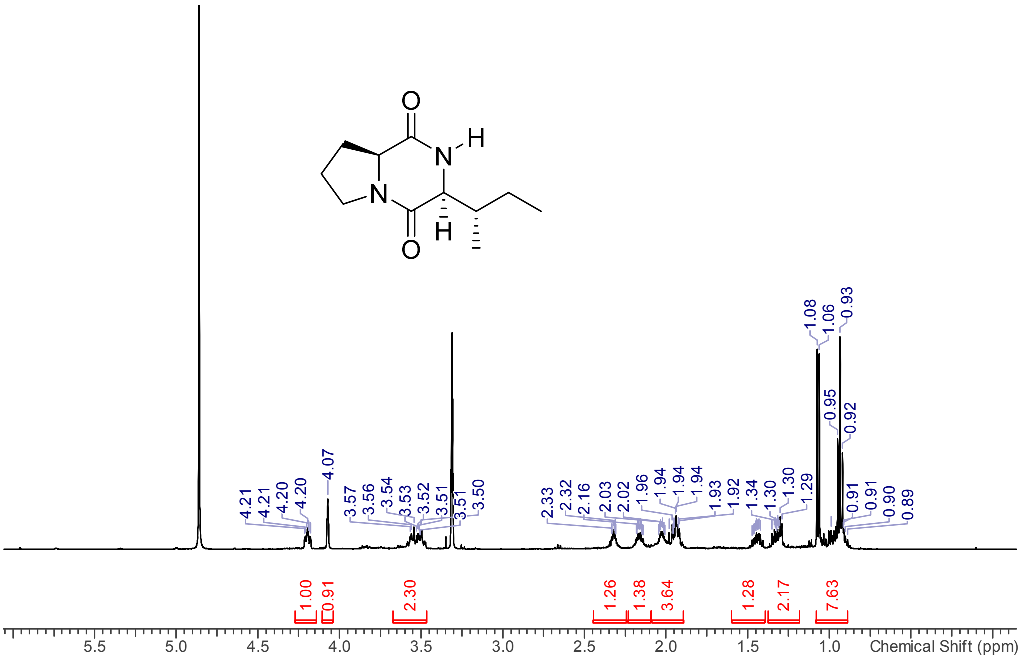

S11. ^{1}H NMR spectrum (MeOH-d_4, 700 MHz) of *cyclo*(*S*-Pro-*S*-Ile) (**3**).

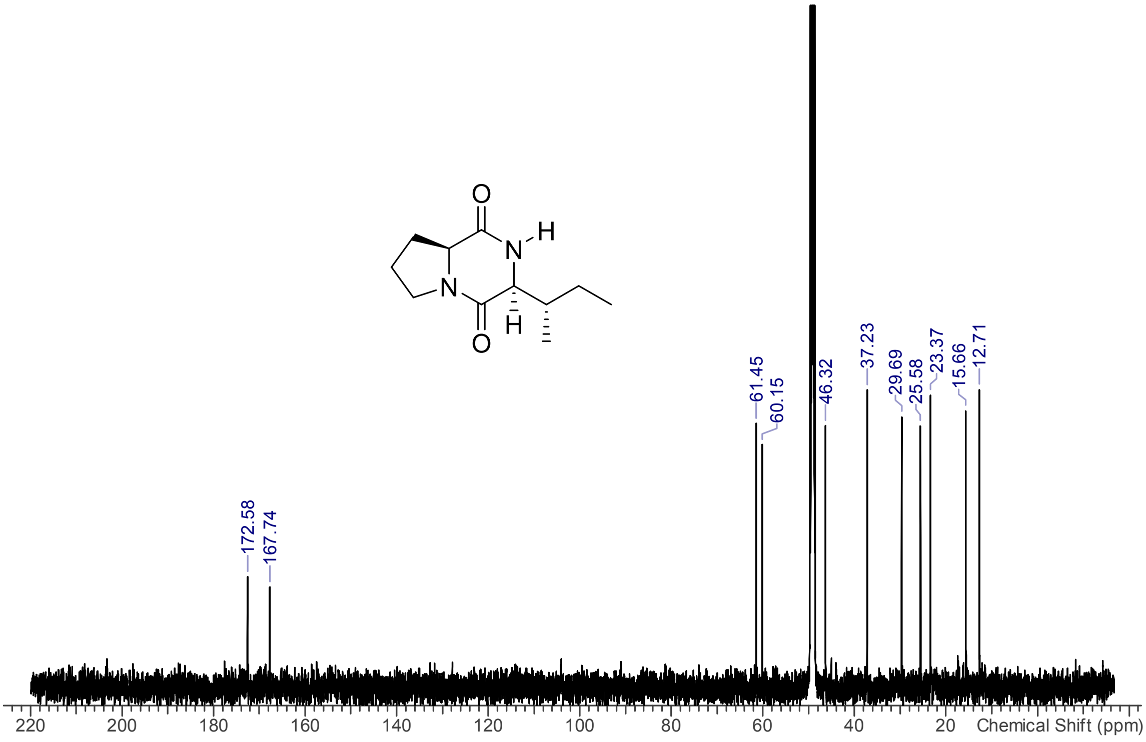

S12. ^{13}C NMR spectrum (MeOH-d_4, 175 MHz) of *cyclo*(*S*-Pro-*S*-Ile) (**3**).

Pedras, M.; Soledade C.; Yu, Y.; Liu, J.; Tandron-Moya, Y.A. *Zeitschrift fuer Naturforschung, C: J. Biosci*. **2005**, *60*, 717–722.

S13. Antimicrobial assay results of compounds **1-3**.

Table S13. Antimicrobial activities of compounds **1-3**.

Strain	Compound 1	Compound 2	Compound 3	Positive control (μg/mL)	
**Bacteria**	MIC (μg/mL)				
Bacillus subtilis DSM 10	66.7	-	-	4.1	Oxytetracyclin
Chromobacterium violaceum DSM 30191	-	-	-	0.4	Oxytetracyclin
Escherichia coli DSM 1116	-	-	-	3.3	Oxytetracyclin
Micrococcus luteus DSM 1790	66.7	-	-	0.2	Oxytetracyclin
Mycobacterium smegmatis ATCC 700084	-	-	-	3.3	Kanamycin
Pseudomonas aeruginosa PA14	-	-	-	1.7	Gentamycin
Staphylococcus aureus DSM 346	66.7	-	-	3.3	Oxytetracyclin
**Fungi**					
Candida albicans DSM 1665	-	-	-	66.7	Nystatin
Mucor hiemalis DSM 2656	66.7	-	-	33.3	Nystatin
Pichia anomala DSM 6766	-	-	-	33.3	Nystatin
Rhodoturula glutinis DSM 10134	-	-	-	33.3	Nystatin
Schizosaccharomyces pombe DSM 70572	-	-	-	33.3	Nystatin
**Cell lines**	Cytotoxicity IC_{50} (μg/mL)				
KB 3.1	14	-		5×10^{-4}	epothilone B
L929	27	-		1.1×10^{-3}	epothilone B

(−) no activity

S14. Cytotoxicity assay results of compounds **1**.

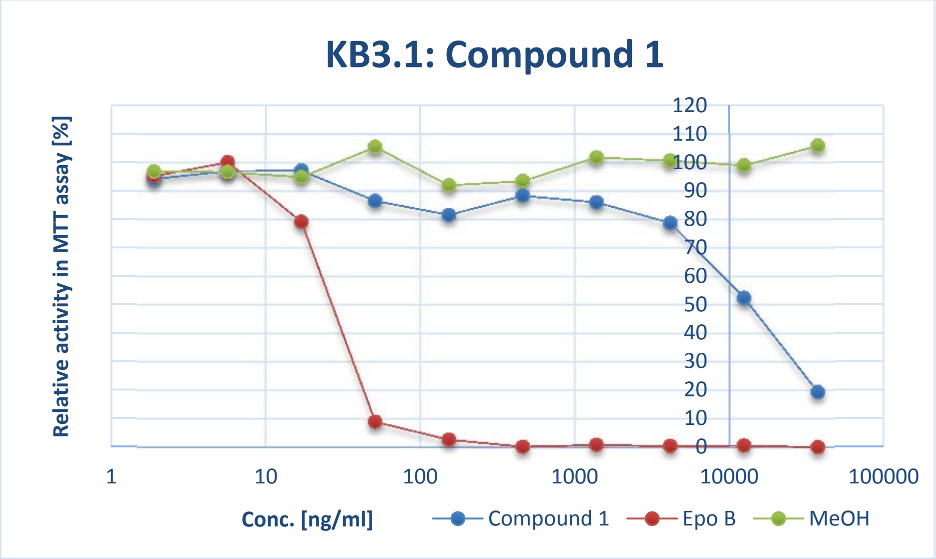

A

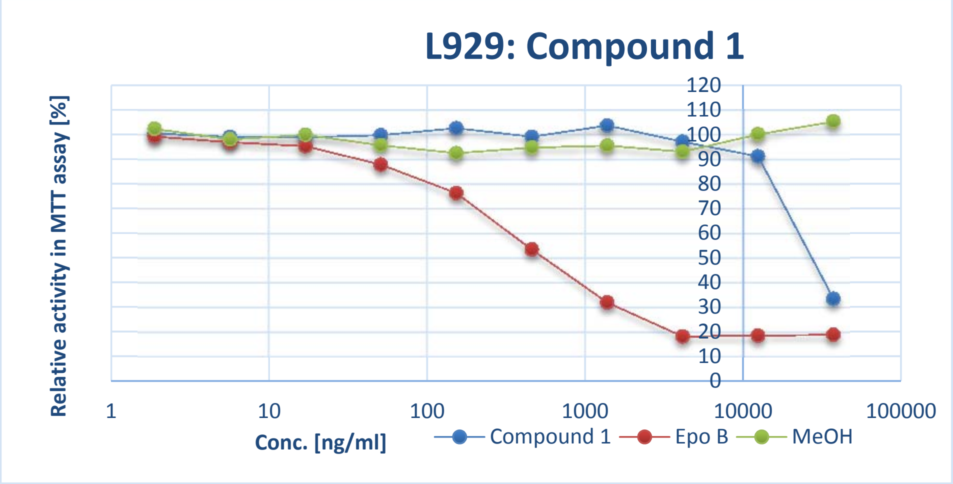

B

Figure S14. Proliferation assay. Graph of MTT assay after 5 days of incubation. *In vitro* cytotoxicity (IC_{50}) of compounds **1** was determined against HeLa (KB-3.1) (A) and mouse fibroblast L929 (B) cell lines. Epothilone B was used as the positive control while methanol was used as the negative control.

S15. Biofilm inhibitory activity of compounds **1-3.**

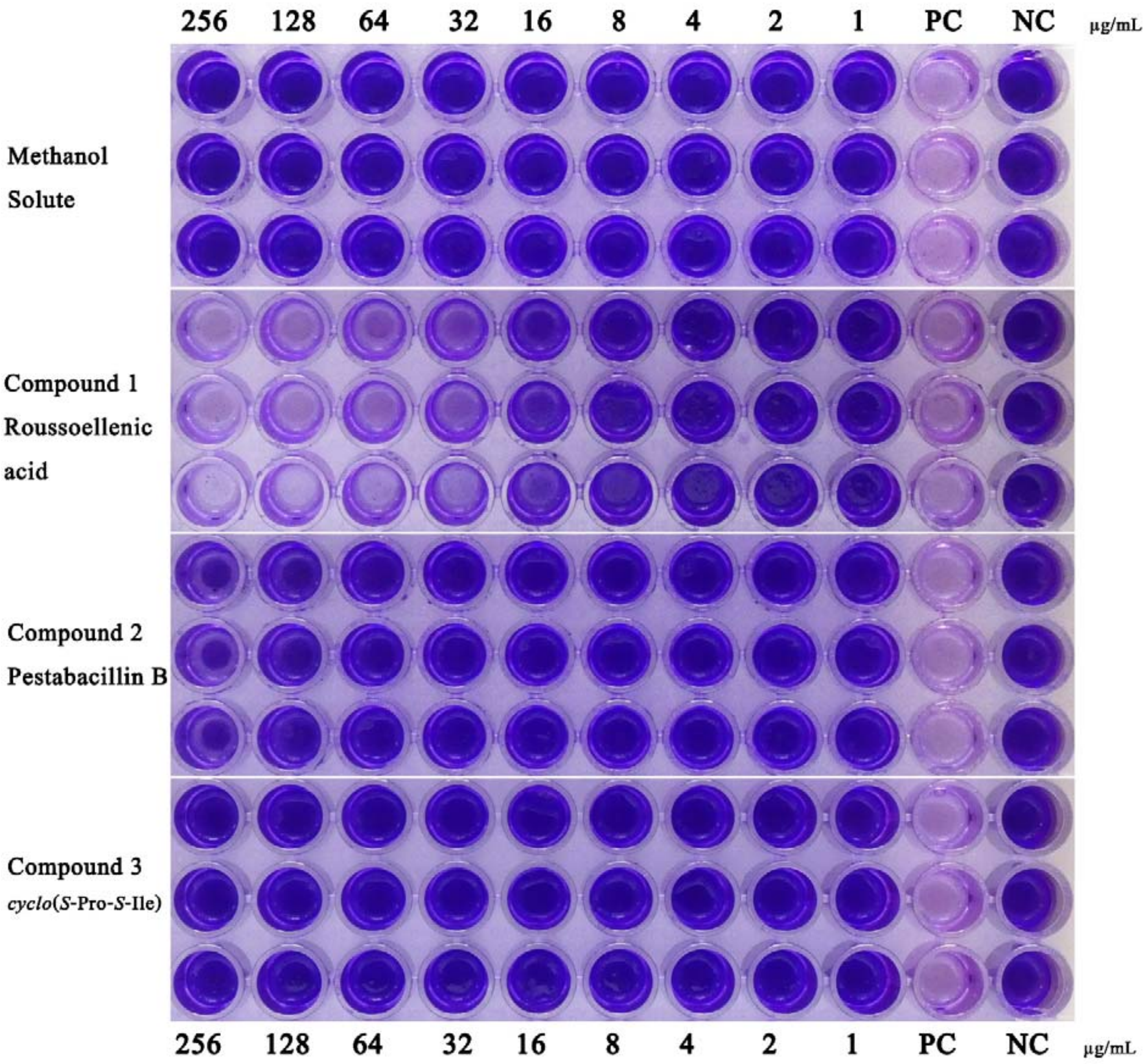

Figure S15. Inhibition of the biofilm formation from *Staphylococcus aureus.* CASO medium containing 4% glucose was used as the negative control and tetracycline (100 µg/mL) was used as the positive control.
